# Non-alcoholic fatty liver disease – a procoagulant condition?

**DOI:** 10.3325/cmj.2021.62.25

**Published:** 2021-02

**Authors:** Lucija Virović-Jukić, Sanja Stojsavljević-Shapeski, Jelena Forgač, Michal Kukla, Ivana Mikolašević

**Affiliations:** 1 Department of Gastroenterology and Hepatology, Sestre Milosrdnice University Hospital Center, Zagreb, Croatia; 2 Department of Internal Medicine, University of Zagreb School of Medicine, Zagreb, Croatia; 3 Department of Endoscopy, University Hospital in Krakow, Krakow, Poland; 4 Department of Internal Medicine and Geriatrics, Jagiellonian University Medical College, Krakow, Poland; 5 Department of Gastroenterology, School of Medicine, University of Rijeka, Rijeka, Croatia; 6 Department of Gastroenterology, Merkur University Hospital, Zagreb, Croatia; 7 University of Rijeka, Faculty of Medicine, Rijeka, Croatia

## Abstract

Non-alcoholic fatty liver disease (NAFLD) is associated with a number of extrahepatic comorbidities and considerable cardiovascular morbidity and mortality, which is possibly related to coagulation changes associated with metabolic syndrome. Coagulation disorders are common in patients with liver disease of any etiology, and here we review possible alterations in coagulation cascade specific to NAFLD. We discuss derangements in the coagulation cascade and fibrinolysis, endothelial dysfunction, and platelet abnormalities as possible culprits for altered coagulation and explore the significance of these changes for potential treatment targets.

Non-alcoholic fatty liver disease (NAFLD) represents a hepatic manifestation of the metabolic syndrome and has recently been termed metabolic-associated fatty liver disease (MAFLD). It is characterized by fat accumulation affecting more than 5% of hepatocytes, which occurs because of insulin resistance and in the absence of secondary causes of liver steatosis. NAFLD includes a spectrum of histological changes with different severity and prognosis, ranging from non-alcoholic fatty liver (NAFL) or simple steatosis to non-alcoholic steatohepatitis (NASH), characterized by inflammation, hepatocyte degeneration, and progressive fibrosis that leads to liver cirrhosis and hepatocellular carcinoma development.

Consequences of NAFLD are not exclusively related to the complications of end-stage liver disease, but significant morbidity and mortality are attributed to cardiovascular and malignant diseases (1). Well-established risk factors for the development of cardiovascular diseases and their complications are various pathophysiologic mechanisms, such as insulin resistance, dyslipidemia, inflammation, oxidative stress, and adipokine imbalance. Recent research, however, has focused on changes in hemostatic process and their contribution to the progression of liver disease and the development of cardiovascular complications of NAFLD and metabolic syndrome.

Coagulation system changes are common in patients with advanced liver disease, since most of the coagulation factors, including fibrinogen, thrombin, and factors V, VII, IX, and X are synthesized in the liver. Additionally, posttranslational modification of coagulation factors also takes place in hepatocytes, and in the setting of hepatocyte injury and liver disease, their function can quantitatively and qualitatively change, leading to hemorrhagic diathesis.

On the other hand, hepatocyte injury can lead to a procoagulant state due to an increased production of inflammation mediators (ie, plasminogen activator inhibitor-1 [PAI-1]) together with altered endogenous coagulation inhibitors (protein C, protein S, and antithrombin) and fibrinolytic factors synthesis, as well as decreased clearance of von Willebrand factor (vWF) synthetized by endothelial cells.

In summary, patients with liver pathology are susceptible to a number of diverse coagulation disorders that result in “rebalanced” hemostasis, potentially leaning toward either hemorrhage or thrombosis depending on the disease etiology and liver injury severity (2,3).

## Hypercoagulation in non-alcoholic fatty liver disease

**Figure 1:**
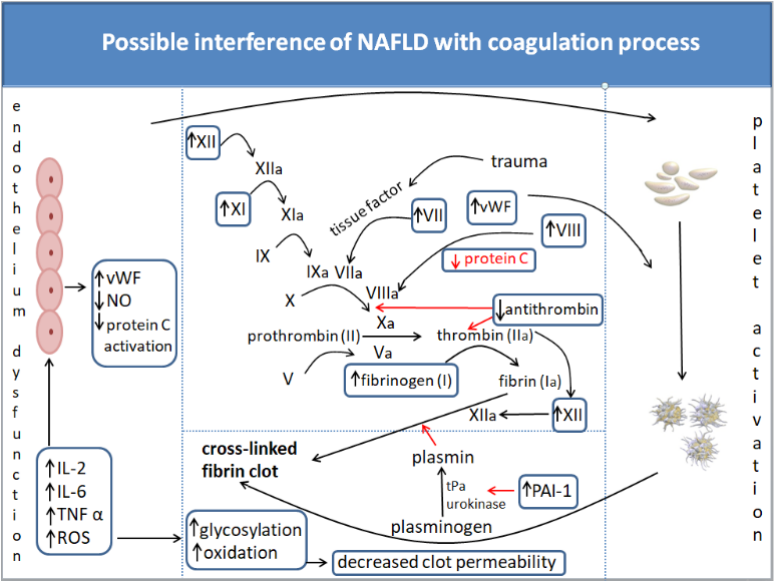
Changes in coagulation factors, platelet activity, fibrin clot properties, and endothelial dysfunction in patients with non-alcoholic fatty liver disease (NAFLD) leading to a procoagulant state. Abbreviations: IL – interleukin; NO – nitric oxide; PAI – plasminogen activator inhibitor; ROS – reactive oxygen species; tPA – tissue plasminogen activator; TNF – tumor necrosis factor; vWF – von Willebrand factor.

Over the last decade, NAFLD has received increased attention, not only as a part of metabolic syndrome, but also as a possible independent contributing factor to a number of disorders, primarily cardiovascular disease and its complications. Because these two entities share a common underlying process, it is sometimes difficult to distinguish whether the changes in coagulation pathways associated with NAFLD reflect the consequences of liver disease *per se* or more probably represent the net result of different processes and components associated with insulin resistance and metabolic syndrome. According to several studies, NAFLD is independently associated with endothelial vascular dysfunction and atherosclerosis, both related to a chronic proinflammatory state that could lead to a prothrombotic state (4). This prothrombotic state seems to be caused by derangements in several components or mechanisms involved in the hemostatic process, including endothelial and platelet dysfunction, alterations in the coagulation cascade, decreased fibrinolytic activity, or a combination thereof (Figure 1) (5). Thrombophilic processes related to liver disease can result in macrovascular incidents, including cerebrovascular and coronary artery disease, deep venous and pulmonary thromboembolism, or splanchnic venous thrombosis, and microvascular changes; the latter process can cause microthrombi formation in the hepatic venules, possibly affecting the course of liver disease (6,7). Hypercoagulable state in cirrhotic patients might induce further hepatic injury by a process known as “parenchymal extinction” (7,8). The underlying mechanism includes the obliteration of hepatic and portal venules by microthrombi, disrupting the normal blood flow and resulting in congestion, local ischemia, and tissue injury (7,8). The consequent hepatocyte apoptosis causes the extinction of the parenchyma, which is replaced by fibrous septa (7). Another possible mechanism involves direct hepatic stellate cells activation mediated by increased thrombin levels and coagulation proteases, even in the absence of intrahepatic thrombosis (8). This model is also applicable to cirrhosis resulting from NAFLD/NASH, as the activation of the coagulation cascade may trigger tissue ischemia and cause a progression of fibrosis in NASH, resulting in hepatic remodeling and cirrhosis development (6). The evidence that prothrombotic state may result in accelerated fibrosis was provided by a faster fibrosis progression in patients with chronic hepatitis C infection and inherited thrombophilia (5).

Conversely, there is increasing evidence that antithrombotic treatment not only prevents thromboembolic incidents in cirrhotic patients, but also potentially slows down liver disease progression. This thesis is supported by a slower progression to decompensated cirrhosis in patients with hemophilia and in patients with compensated liver cirrhosis who were given prophylactic doses of low-molecular weight heparin (5,9).

## Coagulation cascade in non-alcoholic fatty liver disease

Several authors have investigated the alterations in the coagulation cascade associated with NAFLD; however, most of them established the diagnosis of NAFLD using non-invasive methods. Only a minority of the studies involved the use of liver biopsy, which enables the differentiation between various disease stages (simple steatosis, NASH with different stages of fibrosis, or metabolic cirrhosis). Therefore, the analysis is often burdened by the lack of data on the NAFLD stage, presence or absence of inflammation, fibrosis, or cirrhosis. Another challenge are the parameters used to determine the alterations in the coagulation system in clinical practice and in the published studies. Routinely used coagulation tests such as prothrombin time and activated thromboplastin time, or singular pro- and anticoagulant factors measurement, cannot reveal extremely complex interactions that occur in the coagulation cascade *in vivo.* This is of particular importance in cirrhosis, where traditional tests measure only decreased synthesis of coagulation factors and inhibitors, and are not able to determine bleeding diathesis or increased thrombotic risk. Viscoelastic tests represent alternative assays able to evaluate a global hemostatic profile, but are not widely accessible and are therefore underused in clinical practice (10).

Multiple studies implicate a possible relationship between NAFLD and hypercoagulable state. A study enrolling 54 patients with NAFLD diagnosed by proton magnetic resonance spectroscopy found that higher levels of coagulation factors VIII, XI, and XII correlated with liver fat content (11). A study enrolling 273 participants with histologically diagnosed NAFLD reported increased levels of fibrinogen, factor VIII, and vWF factor and decreased levels of antithrombin (12). The results suggest that changes in these coagulation factors correlated with metabolic risk factors, but not with histological changes (12). Significantly increased PAI-1 levels were the only parameter correlating with NAFLD severity defined by NASH activity score (12). A recent study performed by a group from Milan, which enrolled 113 patients with different stages of NAFLD-related liver injury, found a significant procoagulant predisposition, more prominent in patients with more severe liver pathology (ie, metabolic cirrhosis) than in those with simple steatosis (13). Several other studies in patients with NAFLD/NASH found higher levels of factors VII, VIII, IX, XII, vWF, and tissue factor (TF), as well as increased procoagulant microparticles and platelets aggregation, probably related to higher C-reactive protein, PAI-1, and fibrinogen levels (4,6,8,11,14-17).

On the other hand, two recent studies, one enrolling 92 and the other enrolling 68 patients with biopsy-diagnosed NAFLD, also suggested that changes in coagulation factors leading to a procoagulant condition were more likely to be attributed to obesity or insulin resistance than to liver fat content (18,19). Acknowledging the results of these studies, we can assume that the levels of some factors in the coagulation cascade (ie, fibrinogen) are initially increased in NAFLD, but decrease with liver disease progression, resulting in hemorrhagic diathesis, often found in patients with advanced disease (20). In conclusion, data on the coagulation changes in NAFLD are conflicting, and further clinical studies with biopsy established NAFLD diagnosis and staging are eagerly awaited.

## Coagulation in non-alcoholic fatty liver disease cirrhosis

Changes in the coagulation system occur in advanced liver disease regardless of etiology (21-23). Procoagulant and anticoagulant levels decrease in cirrhosis, resulting in a rebalanced hemostasis (24). However, this balance is less stable than in physiological conditions, and either hemorrhage or thrombosis can easily prevail, depending on the risk factors in an individual patient. The most common manifestation of procoagulant imbalance is portal vein thrombosis, a condition of multifactorial origin resulting from a combination of local and systemic alterations (25,26). Local factors contributing to portal vein thrombosis include changes in the liver architecture consequent to fibrosis, with reduced blood flow and endothelial activation, combined with systemic changes resulting from increased levels of procoagulant factors and reduced levels of coagulation inhibitors (27-31).

The particular contribution of NAFLD to the changes in coagulation cascade in advanced liver disease is less well documented, mainly because it is often difficult to establish the diagnosis of NAFLD as the underlying disease in cirrhosis and because studies evaluating coagulation disorders in patients with NAFLD do not distinguish between various disease stages.

Several studies investigating the impact of portal vein thrombosis on the outcomes of patients with cirrhosis found a similar proportion of patients with and without portal vein thrombosis among patients with NASH etiology, although the total number of NASH patients in the cohorts was very small (32-34).

In a study by Berry et al (35), analyzing data from the United Network for Organ Sharing registries, NASH was the etiologic factor for cirrhosis in 12.3% of patients with portal vein thrombosis and in 7.4% of patients without portal vein thrombosis. This could suggest a procoagulant effect of NASH even in the cirrhotic stage, although statistical and clinical significance of this finding has not been addressed by this study (35). Recently, Stine et al (36) have found that in hospitalized patients with cirrhosis, NASH etiology was associated with almost 2.5-fold increased risk of deep venous thrombosis, suggesting that cirrhosis resulting from NAFLD might be particularly prothrombotic.

## Endothelial dysfunction

Endothelial cells represent a barrier between the blood circulating through the vessels and the surrounding tissues. They are crucial for many processes and vessel functions, including the regulation of vascular tone, blood clotting, and inflammation. Endothelial cells normally prevent blood clotting and thrombus formation within the intact vessels, thus allowing a normal flow of fluid and substances into and out of tissues. On the other hand, a vessel injury or exposure to certain proinflammatory cytokines activates coagulation cascade to prevent hemorrhage and further tissue damage. Endothelial cells modulate the prothrombotic and procoagulant processes by controlling the release and function of numerous coagulation and fibrinolytic factors (ie, thrombomodulin expression, activation of protein C/protein S pathway, inactivation of activated thrombin by antithrombin, release of tissue factor pathway inhibitor [TFPI], etc), by decreasing platelet adhesion and activation (negatively charged heparan sulfates, release of prostacyclin and nitric oxide), by promoting platelet disintegration once the thrombus is formed, and by vasodilation (37). Since inflammation and an increased influx of inflammatory cytokines causes dysfunction of endothelial cells and contributes to cardiovascular morbidity and mortality, it was of interest to investigate whether liver inflammation, as observed in NAFLD, could provoke the same effect by promoting hypercoagulable state driven by endothelial injury (37). Bilgir et al (14) found increased levels of both plasma vWF and TFPI in patients with NAFLD, while other coagulation activators or inhibitors, such as PAI-1 and thrombomodulin, did not significantly change. According to authors, these findings provide evidence for endothelial injury in NAFLD, which can trigger atherosclerotic process and lead to adverse cardiovascular events, thus confirming a close connection of NAFLD and cardiovascular disease (14).

Some studies have addressed the importance of perivascular adipose tissue, which, similar to visceral adipose tissue, releases free fatty acids and numerous adipocytokines, such as leptin, adiponectin, resistin, interleukin (IL)-6, PAI, and tumor necrosis factor-α (TNF-α) (38). Although the exact mechanisms how these bioactive molecules diffuse to the vessels is not known, the process occurs in a paracrine manner and via *vasa vasorum*, thus contributing to cardiovascular diseases (39). Adiponectin acts through numerous mechanisms, such as nitric oxide (NO) production, blockage of adhesion molecule expression on endothelial cells, and formation of neointima after arterial injury (40-42). In this way, it favorably affects arterial hypertension, thrombosis, and endothelial dysfunction, and is consequently decreased in perivascular adipose tissue in obesity (43,44). Hyperleptinemia, on the other hand, as a consequence of leptin resistance in obesity and metabolic syndrome, decreases NO release and promotes reactive oxygen species (ROS) formation, which worsens the hypercoagulable state and coronary artery disease (45). Resistin also acts unfavorably and promotes the progression of coronary artery disease by mechanisms similar to those of leptin, namely by reducing endothelial NO production and increasing ROS (46). It also has a proatherogenic effect and promotes restenosis and vascular remodeling by increasing adhesion molecule expression on endothelial cells, as well as cell proliferation through protein kinase B (Akt) pathways (47,48). Furthermore, several studies have confirmed a positive correlation between serum resistin levels and coronary artery disease (49).

## Platelet abnormalities

A recent large meta-analysis combining data from eight cross-sectional and cohort studies and including 842 participants with NAFLD found that NAFLD patients compared with controls had significantly increased mean platelet volume (MPV) (50). A study enrolling 100 patients with biopsy-proven NAFLD showed a significant stepwise increase in MPV levels from participants with normal histology through patients with simple steatosis to those with NASH; MPV also significantly correlated with the histological features of NASH, including steatosis, inflammation, ballooning, and fibrosis (51). MPV is a simple and inexpensive routine laboratory test, used for measuring the platelet size, an indirect marker of platelet activity and a potential indicator of prothrombotic milieu (50). The pathophysiological mechanism behind this observation is not fully elucidated, but is probably based on the inflammatory process underlying NAFLD, which increases various inflammatory mediators, such as IL-1, IL-6, and TNF-α (50). These proinflammatory cytokines alter the platelet size, with larger platelets consequently secreting more granules and prothrombotic factors (52,53). Increased MPV also increases the risk of acute myocardial infarction and stroke, and is associated with higher stent restenosis and poor outcomes after stroke (50).

## Decreased fibrinolysis and changes in fibrin clot structure

Several studies have observed an association between changes in the fibrin clot structure with reduced permeability and an increased risk of thrombosis (19,54). Patients with cirrhosis irrespective of etiology have significantly decreased fibrin clot permeability, although they have reduced plasma fibrinogen levels (19,54). However, before the development of cirrhosis the levels of prothrombotic factors, including fibrinogen, are increased in patients with NAFLD, and they directly correlate with underlying histology and severity of the disease (51). The procoagulant changes in the fibrin clot structure were associated with oxidative modifications of the fibrinogen molecule. On the other hand, decreased fibrin clot permeability is further promoted by enhanced fibrinogen glycation in patients with diabetes, so it is unclear if prothrombotic changes in the fibrin clot structure are driven by obesity and metabolic syndrome-associated disorders or by liver disease, considering a significant overlap between NAFLD patients and those diagnosed with metabolic syndrome (19).

## Possible treatment options

Considering all the derangements in the coagulation process possibly associated with NAFLD, whether related directly to liver disease itself or indirectly through inflammatory processes and metabolic disorders as seen in the metabolic syndrome, a number of potential targets for treatment could be identified.

Since they were recognized as modulators of systemic inflammatory processes, omega-3 polyunsaturated fatty acids (ω-3 PUFA) and their effect on NAFLD/NASH progression were assessed by several meta-analyses and systematic reviews (55,56). Although ω-3 PUFA supplementation may improve liver fat content, possible beneficial effects on necroinflammatory changes as seen in NASH are less clear (55,56). However, ω-3 PUFAs have an established effect on platelet function that might indirectly ameliorate histologic progression of liver disease by preventing thrombotic incidents and the resulting tissue ischemia that promotes liver remodulation (57). They may affect platelet activation through platelet membrane changes, which increases negative membrane surface charge and prolongs the bleeding time (58). However, the exact benefit of ω-3 PUFA supplementation on liver disease progression is currently unknown, and more studies are needed to clarify their role (59).

The adverse effect of procoagulant state on fibrosis and cirrhosis progression in terms of venous microthrombi and resulting hepatic remodeling suggests it would be prudent to evaluate possible benefits from anticoagulation and anti-aggregation therapy in patients with NAFLD. There is insufficient data on the subject, but Villa et al (60) found a lower rate of liver-related complications in cirrhotic patients treated with low molecular weight heparin. Although hepatitis C virus infection and alcohol were the most common etiologic factors for cirrhosis and there were only two NASH patients in the enoxaparin and the control group, treatment with low-molecular weight heparin was found to possibly delay hepatic decompensation and offer survival benefit to the treated patients (60).

Moreover, due to a substantial overlap between patients with NAFLD and metabolic syndrome, anticoagulation and anti-aggregation therapy could beneficially affect not only liver-related complications but could also reduce the great burden of cardiovascular disease morbidity and mortality caused by hypercoagulable and prothrombotic changes associated with obesity and insulin resistance (59).

## Conclusion

Despite the research efforts made, substantial evidence regarding the link between NAFLD and coagulation disorders is still missing, which could partly be explained by inadequacy of tests used to assess these disorders. Larger population-based studies with a careful selection of methods and factors studied are needed to clarify the complex alterations in the coagulation process occurring in NAFLD *in vivo*, their interactions, and clinical outcomes.

Furthermore, a close association of NAFLD and metabolic syndrome makes it hard to distinguish and quantify the extent to which both entities independently contribute to procoagulant state. However, growing evidence suggests that NAFLD, and especially its necroinflammatory form – NASH, may significantly and independently contribute to procoagulant and prothrombotic state, irrespective of the presence of the confounding factors related to the presence of insulin resistance and metabolic syndrome. Nevertheless, there is an undeniable evidence that the two conditions together potentiate adverse outcomes in terms of cardiovascular complications, and that changes in coagulation profiles in patients with NAFLD affect the rate and severity of their liver disease progression. These results emphasize the potential clinical benefits of the assessment of the prothrombotic and procoagulant risk factors in NAFLD patients and could pave the way for new therapeutic approaches.
